# One-year outcomes of faricimab for neovascular age related macular degeneration with OCT angiography: focus on resistant and refractory cases

**DOI:** 10.1007/s10792-025-03717-w

**Published:** 2025-08-19

**Authors:** Alessandra Scampoli, Matteo Mario Carlà, Giulia Grieco, Lorenzo Governatori, Roberta Catalani, Stanislao Rizzo, Tomaso Caporossi

**Affiliations:** 1 Present Address: Vitreoretinal Surgery Unit, Isola Tiberina Hospital - Gemelli Isola, Rome, Italy; 2 Ophthalmology Department, Fondazione Policlinico Universitario A. Gemelli, IRCCS”, 00168 Rome, Italy; 3 Ophthalmology Department, Catholic University “Sacro Cuore”, Rome, Italy

**Keywords:** Age-related macular degeneration, Anti-vascular endothelial growth factor, Faricimab, Resistant nAMD, Treat-and-extend, OCT angiography

## Abstract

**Purpose:**

To investigate the 12-month effectiveness and safety of intravitreal faricimab (IVF) in patients with neovascular age-related macular degeneration (nAMD) resistant to previous anti-VEGF treatment.

**Methods:**

Prospective, monocentric study including consecutive patients with resistant/refractory nAMD switched to IVF between July 2023 and November 2024. Primary endpoints were safety, best corrected visual acuity (BCVA), central subfield thickness (CST), and subfoveal choroidal thickness. Secondary endpoints included changes in optical coherence tomography (OCT) and OCT angiography biomarkers: fluid prevalence, pigment epithelial detachment (PED) height, and vascular densities. All patients received four monthly loading doses of faricimab, with subsequent treat-and-extend regimen.

**Results:**

The study included 30 eyes of 30 patients. Mean follow-up was 14.2 ± 1.9 months and no adverse events were reported. BCVA significantly improved from 0.77 to 0.62 LogMAR at the end of the study period (p = 0.009), with 67% of eyes showing stable vision. CST significantly decreased from baseline (-57 μm on average, *p* < 0.001), along with PED height which showed its main decrease during the loading phase. Forty-seven percent of eyes achieved complete macular dryness at week 16, with significant reduction in terms of subretinal fluid (SRF) and intraretinal fluid (IRF) prevalence. At the end of the study, 90% of patients achieved treatment intervals of at least q8w, with 27% of eyes being on q12w. Finally, no changes in superficial/deep vessel densities were observed.

**Conclusion:**

Faricimab demonstrated efficacy and safety in refractory/resistant nAMD, with significant improvements in structural outcomes and stable/improved visual acuity. Extended treatment intervals suggest a potential reduction in treatment burden.

## Introduction

Neovascular age-related macular degeneration (nAMD) is a primary global contributor to significant visual impairment among the elderly.^1^ The development of macular neovascularization (MNV) results from disrupted equilibrium between pro- and antiangiogenic factors, leading to increased synthesis of pro-angiogenic mediators.^2^ Intravitreal anti-vascular endothelial growth factor (anti-VEGF) therapy has become the backbone of nAMD therapy, though some patients may exhibit treatment resistance, suboptimal response, or develop tachyphylaxis, necessitating repeated injections.^3^

Resistance mechanisms include activation of alternate angiogenic pathways independent of VEGF signaling, chronic retinal inflammation promoting angiogenesis and fibrosis, and dysregulation of Tie-2 signaling essential for vascular integrity.^4,5^ Faricimab (Vabysmo; Roche/Genentech, Switzerland) is a dual inhibitor of VEGF and Ang-2, approved by the FDA (February 2022) and EMA (October 2022).^6^ This bi-specific, humanized IgG monoclonal antibody represents the first dual-pathway inhibitor for intraocular use.^7^

Clinical Phase 3 studies, TENAYA and LUCERNE, demonstrated non-inferiority to aflibercept in treatment-naïve nAMD, with over 45% of patients achieving 16-week intervals (q16w), potentially reducing injection burden and improving long-term treatment sustainability.^6,8,9^

While recent real-world data shows favorable results in treatment-naïve eyes,^10–13^ limited data exists on faricimab efficacy in patients switched from other anti-VEGF medications.^14–17^ Thus, we investigated the effectiveness and safety of IVF in patients with nAMD showing partial response or resistance to other anti-VEGF agents after at least 12 months of follow-up.

## Materials and methods

This prospective monocentric interventional research, conducted at Ospedale Isola Tiberina-Gemelli-Isola, Rome, Italy between July 2023 and November 2024, included all consecutive patients with resistant nAMD who were switched to intravitreal faricimab (IVF). This investigation adhered to the principles of the Helsinki Declaration, and the inquiry was approved by the Ethical Committee of the Catholic University of the Sacred Heart. A comprehensive elucidation of the research procedure was presented, and informed consent was obtained from all participants.

We included eyes with nAMD who were already undergoing an active treatment with other anti-VEGFs following a treat-and-extend (TAE) regimen. Criteria for resistance or refractoriness to previous treatment were: 1) eyes that had undergone 6 or more anti-VEGF IVIs in the 12 months or 4 or more IVIs in the 6 months before baseline; 2) Persisting, exacerbating, or repeated intraretinal fluid (IRF) or subretinal fluid (SRF) shown on spectral-domain optical coherence tomography (SD-OCT) scans after three consecutive intravitreal injections. Only patient who had at least 12 months of follow-up subsequent to the transition from other anti-VEGFs to IVF were included. Both type I, type II, type III and mixed MNV were included.

The exclusion criteria included: eyes that had undergone an ocular procedure other than anti-VEGF treatment within 6 months of baseline or during the study period (e.g., cataract surgery, glaucoma filtration surgery, pars plana vitrectomy), 2) the patient had a concomitant retinal vascular or tractional disease (e.g., diabetic retinopathy, vascular occlusion, vitreoretinal interface disorder), an history of vitrectomy, uveitis or intraocular inflammation, and history of severe ocular conditions before the enrollment; 3) the patient was lost to intended/scheduled follow-up during the study period or 4) valuable OCT imaging was not achievable due to media opacity (e.g. corneal disease, cataract).

At baseline, demographics and medical history, as well as the number of previous IVIs, were documented. During each visit, patients received a slit-lamp examination, dilated fundoscopy, intraocular pressure (IOP) measurement, and assessment of best corrected visual acuity (BCVA), evaluated using Snellen charts and successively converted to LogMAR for statistical purposes. Potential adverse effects were examined and delineated. SD-OCT and OCT angiography (OCTA) analyses were conducted at baseline and at each follow-up time point. For statistical analysis, we identified five clinically relevant timepoints for the evaluation of morpho-functional parameters: V0 (baseline, treatment initiation decision); V1 (week 12, completion of loading dose and assessment of initial response); V2 (week 16, one month post-loading dose, critical evaluation point for determining initial extension feasibility); V3 (first follow-up after initial extension, assessment of interval tolerance); V4 (week 48–52, one-year milestone for long-term efficacy evaluation); V5 (most recent follow-up, final treatment response assessment). These timepoints were selected to align with standard clinical decision nodes in treat-and-extend protocols.

All patients underwent a loading phase therapy which included four monthly injections of 6 mg faricimab. During the maintenance phase, the injection interval was extended by 4 weeks upon establishing a fully dry macula; conversely, it was shortened by 4 weeks if the criteria was not fulfilled. A dry macula was defined by the lack of intraretinal, subretinal, or sub-RPE fluid, accompanied by absent hemorrhaging. The treatment interval at the end of the study was documented and collected.

### OCT/OCTA data collection

OCT and OCTA imaging were conducted with the RTVue XR Avanti (Optovue Inc, Fremont, California, USA). Scans with subpar indices (< 7/10) resulting from considerable lens opacities, frequent eyelid blinking, severe motion artifacts, and other variables were discarded.

High-definition fovea-centered Macula Cross and HD Raster images were obtained to evaluate OCT biomarkers. Furthermore, the RetinaCube program was used to automatically compute the central subfield thickness (CST), defined as the mean retinal thickness inside a 0.5-mm radius circle at the foveal center. OCTA images were obtained with the AngioVue Retina program, which automatically evaluates the vascular density (VD) of the superficial and deep capillary plexuses (SCP, DCP) inside the central 1-mm sector of the Early Treatment Diabetic Retinopathy Study (ETDRS) maps. Every OCTA picture was subjected to motion correction and 3D projection artifact elimination techniques to enhance image quality.

The whole macula was examined using volumetric scan OCT images to evaluate the presence of sub-retinal fluid (SRF), intra-retinal fluid (IRF), or sub-RPE fluid. The measurement from Bruch's membrane to the sclero-choroidal border at the fovea was used to determine subfoveal choroidal thickness (SFCT). The maximum height of the pigment epithelial detachment (PED) was assessed from the retinal pigment epithelium (RPE) to Bruch's membrane after scan-by-scan analysis of RetinaCube images.

Two impartial masked retinologists (A.S., G.G.) assessed SD-OCT and OCT-A images, with any disagreements resolved by a third observer, T.C. The follow-up mode of the Optovue software was used to guarantee accurate centration and consistency of OCT images. Additionally, a manual verification was conducted separately by two observers at each follow-up. Segmentation problems were manually rectified as needed using the integrated software.

The main endpoints of this research were safety, resolution of retinal fluid, changes in mean BCVA, CST and SFCT over the follow-up period. We assessed changes in OCT biomarkers (fluid prevalence, PED height) and vascular densities (SCP and DCP) as secondary outcomes during the therapy.

## Statistical analysis

Statistical analysis was conducted using GraphPad PRISM Software (Version 10.3; GraphPad, La Jolla, CA). The Shapiro–Wilk test was employed to assess the normality of the sample, with a p-value greater than 0.05 supporting the null hypothesis of normal distribution. Mann–Whitney U test was utilized with a 95% confidence interval (CI) to compare continuous variables. Two-way analysis of variance (ANOVA) with Dunnett correction was used to evaluate changes in BCVA and OCT parameters during follow-ups. Correlation analysis was performed using the Spearman coefficient. χ2 test and Fisher’s exact test for contingency analysis were conducted where appropriate. Multivariate regression analysis was performed to identify independent predictors of final visual outcomes. Mean ± standard deviation (SD) was used to present quantitative data, and a p < 0.05 was deemed as statistically significant.

## Results

This cohort included 30 eyes from 30 individuals with resistant/refractory AMD, with an average age of 79.4 ± 5.9 years and a 12/18 M/F ratio. Prior to faricimab switch, patients underwent a mean of 16.3 ± 7.2 anti-VEGF injections, with most recent mean treatment interval of 5.9 ± 2.4 weeks. The majority of eyes (21 eyes; 70%) were switched from previous aflibercept, while 9 eyes (30%) were switched from brolucizumab. Regarding MNV subtype, 23 eyes (77%) showed a type 1 MNV, while 7 eyes (23%) showed a mixed MNV morphology. All patients were followed for at least one year, with a mean follow-up of 14.2 ± 1.9 months from baseline.

**Fig. 1:**
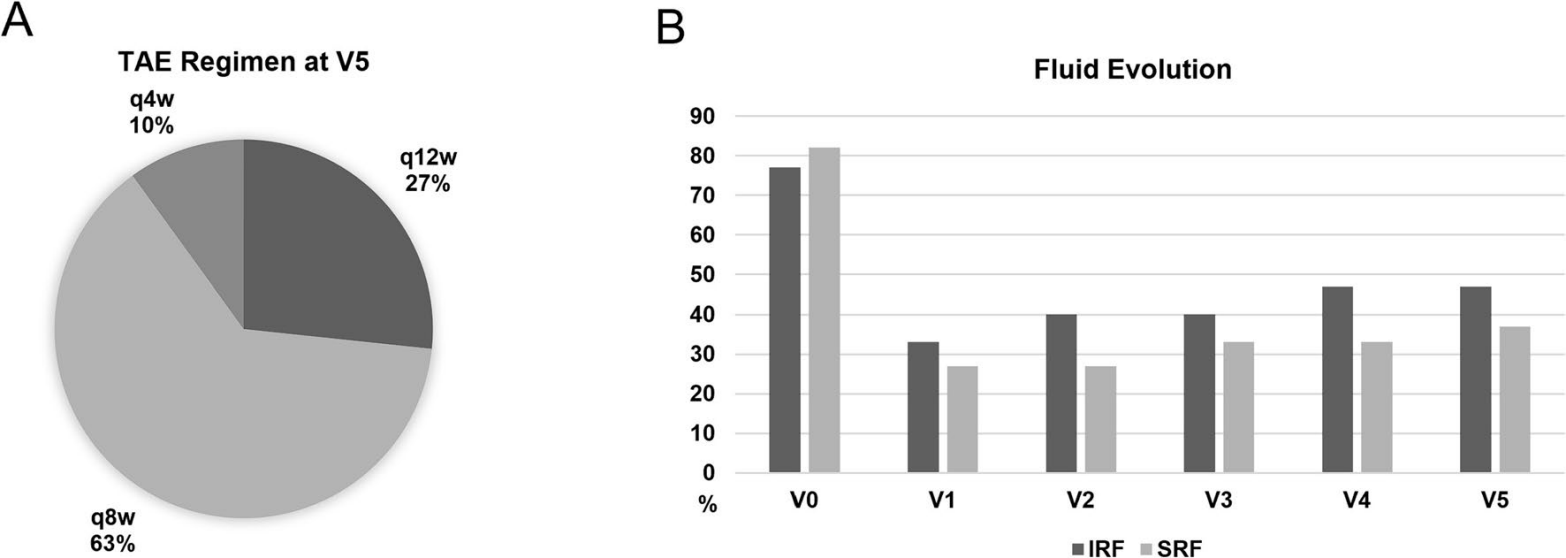
**A** Prevalence of different intervals between injections in the treat-and-extend (TAE) regimen, at the end of the study period; **B** Prevalence of intraretinal and subretinal fluid (IRF and SRF) throughout the follow-ups, with the latter showing a better response to faricimab treatment, even if the reduction was significant for both. During the treat-and-extend regimen (V3-V5), a slight increase was highlighted in terms of fluid prevalence in both sub-localizations

Mean number of IVFs was 9.6 ± 2.2 per eye, with an average interval of 8.7 ± 2.4 weeks at the end of the study period. At the most recent visit, 8 eyes (26.7%) were on a q12w regimen, 19 eyes (63.3%) on q8w and 3 eyes (10.0%) were still on q4w. None of the eyes experienced post-injection adverse events or intraocular inflammation (IOI). (Fig. 1).

At week sixteen, one month after the end of the loading phase, 14 eyes (46.7%) showed a completely dry macula on OCT. These 14 eyes underwent significantly fewer injections overall throughout the course of the study period (8.7 ± 1.1 vs. 10.5 ± 1.9, p = 0.03), with a longer planned injection interval at the final visit, compared to patients with residual fluid at week 16 (11.1 ± 1.4 vs. 6.3 ± 3.5, p = 0.002). At V5, 9 eyes (30.0%) were still showing a dry macula: 7 eyes were on q12w and 2 eyes on q8w regimen.

### Functional results

The BCVA showed a significant increase during the study period (p = 0.009). Baseline and follow-ups BCVA values (compared to baseline) were: 0.77 ± 0.51 LogMAR at V0, 0.49 ± 0.52 LogMAR at V1 (p = 0.001), 0.49 ± 0.54 LogMAR at V2 (p = 0.002), 0.55 ± 0.31 LogMAR at V3 (p = 0.008), 0.61 ± 0.54 LogMAR at V4 (p = 0.02), and 0.62 ± 0.54 at V5 (p = 0.04). (Fig. 1) Overall, 8/30 eyes (26.7%) showed visual improvements during the study period, with 5 eyes (16.7%) exhibiting an improvement of 0.3 LogMAR or more. The majority of eyes (20/30, 66.7%) showed stable visual outcomes, while 2 eyes (6.1%) showed a 0.1 LogMAR decline. One of these two eyes already had a perifoveal RPE tear at baseline showing poor visual results, while the other eye was still on q4w regimen at the end of the follow-up period.

Overall, final visual outcomes did not correlate with thicker CST at V0 (ρ = -0.14; p = 0.29). Conversely, baseline BCVA was a significant predictor final BCVA (ρ = 0.88; p = 0.0001). Multivariate analysis revealed that baseline BCVA remained the strongest predictor of final visual outcomes (β = 0.82, p < 0.001), while baseline CST, PED height, and MNV subtype showed no significant correlation with final BCVA (all p > 0.05).

### Structural and microvascular evolution

Mean CST showed the most important decrease during the loading phase: from 401 ± 225 μm at V0 to 320 ± 227 μm at V1 (mean difference -82 μm, 95% CI 31 to 132 μm; p = 0.0008). Successively. CST values showed stability through the follow-ups: 333 ± 237 μm at V2, 317 ± 218 μm at V3 and 329 ± 188 μm at V4, showing a slight increase at V5 (344 ± 205 μm), but still a significant drop compared to baseline (mean difference -69 μm, 95% CI 39 to 99 μm; p = 0.001). Conversely, SFCT did not show significant changes during the study period ranging from 213 ± 98 μm at V0 to 200 ± 73 μm at V5 (omnibus p = 0.05).

Regarding fluid analysis, 23 eyes (76.7%) showed IRF at V0, compared to 10 eyes (33.3%) at V1, 12 eyes (40%) at V2 and 14 eyes (46.7%) at V5. Conversely, 22 eyes (67.7%) showed the presence of SRF fluid at baseline, reducing to 8 eyes (27%) at V1 and V2. At the end of the study period, 11 eyes (36.7%) still showed the presence of SRF. Contingency analysis confirmed significant reduction of either IRF presence (p = 0.05) and SRF presence (p = 0.009).

**Fig. 2:**
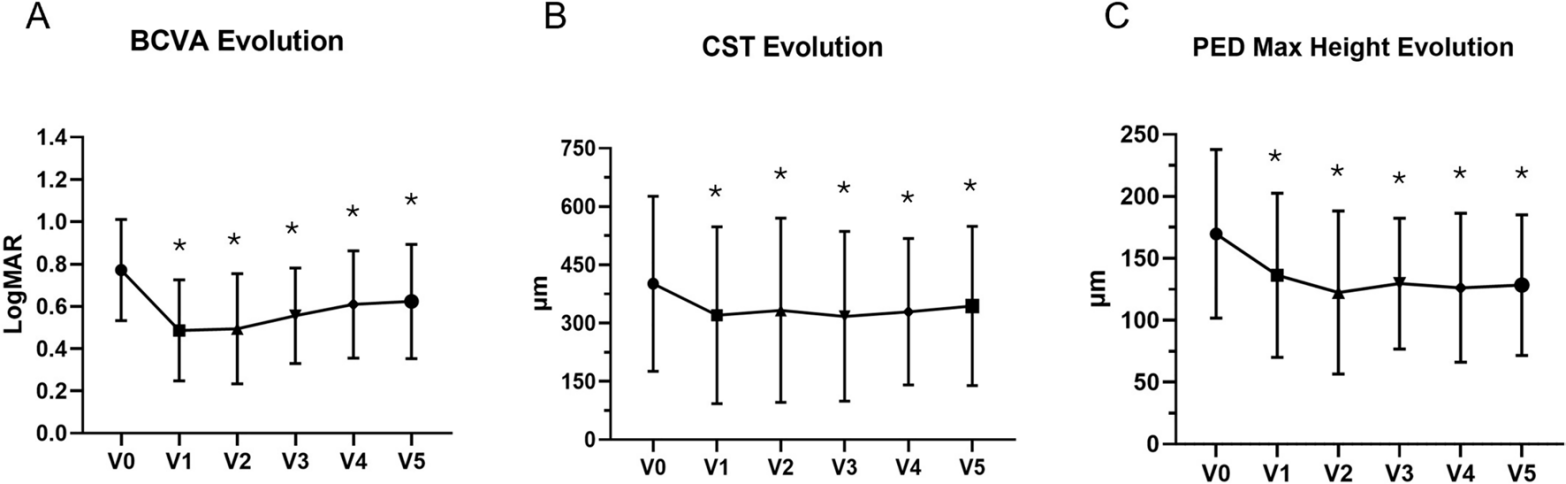
Evolution of functional parameters and OCT biomarkers. **A** Visual improvements during follow-ups; **B** Central subfield thickness (CST) showed a significant improvement starting from V1 and being stable during the study period; **C** Pigmented epithelium detachment (PED) maximum height was significantly reduced during the loading phase, reaching its nadir at V2. Asterisks stand for statistical significance

Similarly, the maximum PED height decreased from 170 ± 182 μm at V0, to 136 ± 177 μm at V1 (p = 0.03), 122 ± 167 μm at V2 (p < 0.001), 130 ± 141 μm at V3 (p = 0.02), 126 ± 161 μm at V4 (p = 0.001) and 128 ± 152 μm at V5 (p = 0.004). The maximum drop in terms of PED height from baseline was seen at V2 (mean difference -47 μm, CI 18 to 76 μm), Successively, mean values were stable during the TAE regimen (mean change V2-V5 + 6 μm, CI -13 to + 25 μm, p = 0.77). Graphical representation of structural parameters evolution is visible in Fig. 2.

Subgroup analysis revealed no significant differences in outcomes between type 1 and mixed MNV subtypes (p > 0.05 for all parameters). Moreover, in a post-hoc subgroup analysis, we found no changes in terms of functional and structural parameters when comparing eyes switched from aflibercept and those switched from brolucizumab (all p > 0.05).

OCTA analysis was focused on the evolution of VD in the SCP and DCP of the central ETDRS subfield. In either plexi, VD showed consistent stability throughout the study period. The SCP’s VD was reportedly 37.8 ± 5.4% at V0 and 38.6 ± 7.1% at V5 (omnibus p > 0.05). Similarly. no significant changes were reported in the DCP’s VD compared to baseline (44.4 ± 2.8% vs. 43.5 ± 4.8% at V5), in either follow-up (omnibus p > 0.05).

**Fig. 3:**
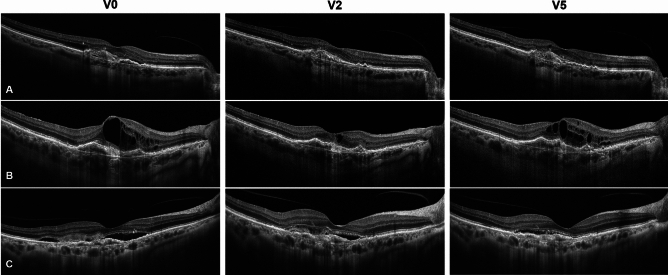
B-scan samples of the response to the loading phase (V0-V2) and successive treat-and-extend (TAE) regimen (V5) with faricimab in 3 eyes. The first case **A** showed a fluid-free macula at V2 and a partial **relapse** of intraretinal fluid (IRF) at V5. The second and third cases **B** and **C** showed partial response to faricimab treatment, but significant reduction in terms of IRF or subretinal fluid (SRF) presence

Samples of B-scan evolution in resistant eyes undergoing IVF are shown in (Fig. 3).

## Discussion

**Fig. 4:**
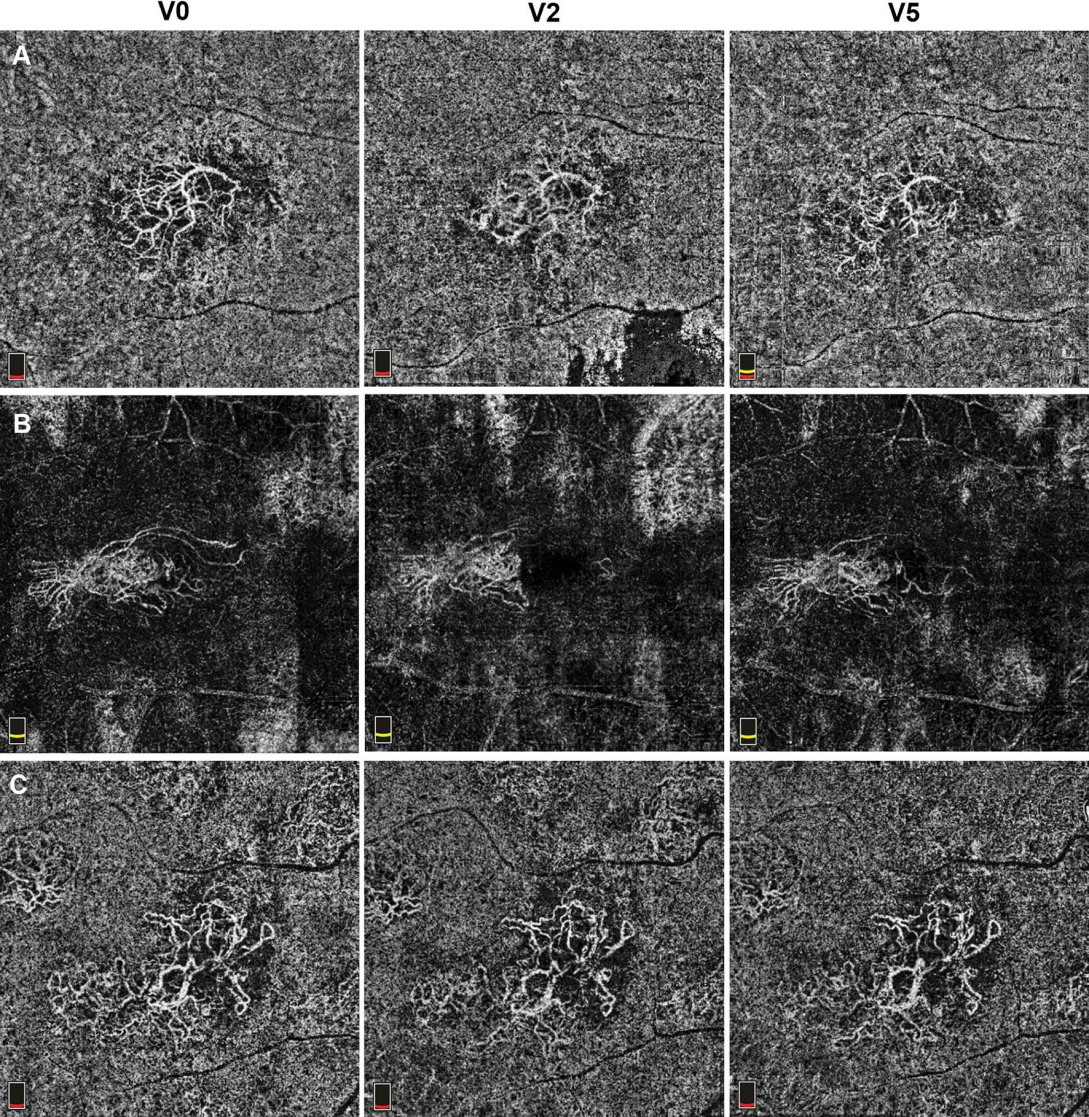
The evolution of macular neovascularization (MNV) networks through different timepoints (V0, V2 and V5) of 3 different patients (A, B, C). Note how the loading phase (interval V0-V2) contributes to reduce the MNV area and the complexity of microvascular architecture, which successively remained almost stable during the follow-up (V2-V5)

Although the new bi-specific antibody faricimab has shown effectiveness in treatment-naïve nAMD patients, its therapeutic benefits in resistant nAMD patients are still uncertain.^18^ The primary objective of this real-world research was to assess the 12-month effectiveness and safety of IVF in patients with nAMD previously showing a partial response or resistance to other anti-VEGF agents (specifically aflibercept and brolucizumab) (Fig. 4).

In this study, the mean BCVA showed a significant improvement from 0.77 to 0.62 LogMAR during the study period, with a strong correlation between baseline and final BCVA. Similar results were shown in Rush et al.’s retrospective interventional case series, which included 54 eyes of 54 subjects with treatment-resistant nAMD. They found that the visual acuity improved from 0.72 logMAR at baseline to 0.59 logMAR at the end of the 12-month study. ^17^ However, other real-world studies have reported mixed results regarding the functional outcomes of faricimab. Tamiya et al. prospectively investigated the efficacy of faricimab in aflibercept-refractory nAMD and observed no BCVA changes 2 months after injection.^18^ Similarly, other reports found no visual acuity changes after switching to faricimab, mainly due to poor baseline vision reflecting neuroanatomical damage from treatment-refractory nAMD.^16,19–21^ Notably, in our series, 67% of eyes showed stable visual function throughout the study period.

At the end of this study, 27% of the eyes were on a 12-week regimen, 63% on an 8-week regimen, and 10% on a 4-week regimen, with 83% of eyes showed prolonged intervals compared to previous treatment with either aflibercept or brolucizumab. These results align with evidence from real-world studies and clinical trials demonstrating that switching to faricimab results in extended treatment intervals. Borchert et al. described a mean treatment interval increase of 1.7 weeks with 57% of eyes having extended treatment intervals at 6 months,^16^ while Machida et al. reported extended dosing intervals of more than 2 weeks in 32.6% of eyes at 6 months.^22^

Focusing on structural effectiveness, this study found that CST showed a significant decrease from baseline (−57 μm on average), with the main CST drop reported at the end of the loading phase. In fact, 47% of eyes showed a completely dry macula at V2. The number of eyes with SRF decreased from 22 (73%) at baseline to 11 (37%) at the end of the study, while the number of eyes IRF decreased from 23 (77%) at baseline to 16 (53%). These results are consistent with previous literature, in which a mean reduction of around 50 μm in terms of central macular thickness at 12 months was reported.^17^ Tamiya et al. observed that 56% of eyes showed reduction or complete absorption of fluid at 2 months,^18^ while Raimondi et al. observed clinical improvement on OCT in 80% of patients.^21^

Schneider et al. found that switching to faricimab in multi-switch eyes resulted in significant decrease in central retinal thickness and PED height even after a single injection.^14^ In our study, the PED maximum height was significantly reduced during the loading phase, reaching its lowest point at 4 months and maintaining this improvement during the TAE phase. These results reflect the progressive increase in PED’s fibrous component during anti-VEGF treatment, leading to a reduction of PED dimensions and a corresponding increase in optical reflectivity.^23^ Borchert et al. reported a statistically significant decrease in mean PED height, from 233 μm at baseline to 188 μm at 6 months.^16^

In our investigation, SFCT remained stable throughout our study period, contrasting with previous meta-analyses suggesting significant choroidal thinning after 3 years of anti-VEGF therapy.^24^ However, our findings of stable SFCT are consistent with other short-term reports on faricimab. Tamiya et al. similarly reported no significant SFCT changes over 2 months in aflibercept-refractory patients.^18^ In addition, Machida et al. identified thinner baseline choroidal thickness as a predictor of successful interval extension after switching to faricimab.^22^ However, literature is still scarce on this subject, and longer-term studies are warranted to assess whether some choroidal changes may be caused by faricimab treatment. Moreover, even if the dual VEGF-A/Ang-2 inhibition may eventually impact some pathways of choroidal architecture, it should be acknowledged that a progressive choroidal thinning may be part of the natural history of the pathology, regardless of the employed anti-VEGF.

The stability of vessel density in both superficial and deep capillary plexuses throughout the study period, despite significant reduction in exudative activity, confirms that faricimab’s therapeutic effect primarily targets pathological vascular permeability rather than vascular structure. Data regarding changes in SCP and DCP vessel density after switching to faricimab in treatment-resistant nAMD are limited, with the majority of published literature showing no significant changes of macular vascular density during anti-VEGF treatment.^25,26^ In agreement with this reports, vessel density of either plexi remained stable throughout the entire study period. However, further studies focusing on this aspect are needed to better understand the impact of faricimab on the retinal vasculature in this patient population. Moreover, even if this study lacked a specific subanalysis on MNV architecture over time, it was recently demonstrated that faricimab was able to reduce area and complexity even in refractory MNVs.^27^

Our study has several limitations. The relatively small sample size (n = 30) limits the statistical power for detecting smaller effect sizes and reduces the generalizability of our findings. Additionally, the absence of a control group makes it impossible to differentiate between natural disease progression and treatment effects. Moreover, even if we employed standardized criteria for treatment switch, a possible selection bias due to physician treatment discretion should be acknowledged. The follow-up period of 14 months, while providing valuable mid-term data, does not allow for assessment of long-term efficacy and safety. Future studies with larger sample sizes, longer follow-up periods, and a randomized controlled design are needed to confirm our findings and further elucidate the long-term effects of faricimab in nAMD.

Our study suggests that faricimab may be effective and safe in refractory and resistant nAMD patients who showed partial response to previous anti-VEGF therapy. While significant improvements in structural outcomes were observed and the majority of eyes showed stable or improved visual acuity, these findings require validation through randomized controlled trials with longer follow-up periods. The potential for extended treatment intervals in this challenging patient population warrants further investigation.

## Data Availability

No datasets were generated or analysed during the current study.
